# Behaviour in childhood is associated with distinct patterns of partnering in adulthood

**DOI:** 10.1192/j.eurpsy.2021.621

**Published:** 2021-08-13

**Authors:** F. Vergunst, Y. Zheng, P. Domond, F. Vitaro, R. Tremblay, D. Nagin, J. Park, S. Cote

**Affiliations:** 1 Public Health, University of Montreal, Montréal, Canada; 2 Psychology, University of Alberta, Edmonton, Canada; 3 Psychoeducation, Umiversity of Montreal, Montreal, Canada; 4 Statistics, Carnegie Mellon University, Pitsburgh, United States of America; 5 Health, Statistics Canada, Ottawa, Canada

**Keywords:** ADHD, Conduct disorder, Disruptive behaviours

## Abstract

**Introduction:**

Childhood behavioral problems are highly prevalent in school-aged children and are associated with poor long-term outcomes. Yet little is known about their association with patterns of partnering in adulthood.

**Objectives:**

To (1) describe patterns of partnering from age 18-35 years in a large population-based sample, and (2) examine the association between childhood behavioural problems and adult partnering patterns.

**Methods:**

Behavioural ratings were prospectively obtained from teachers when children (n=2960) were aged 10-12 years – for inattention, hyperactivity, aggression-opposition, anxiety and prosociality – and linked to their tax return records from age 18-35 years. We used group-based trajectory modelling to identify distinct trajectories of partnering (married or cohabitating) and multinomial regression models to examine the association between childhood behaviour and trajectory group membership.

**Results:**

Five distinct trajectories of partnering were identified: early-partnered (n=420, 14.4%), mid-partnered (n=620, 21.3%), late-partnered (n=570, 19.2%), early-separated (n=460, 15.5%), and delayed-or-unpartnered (n=890, 30.0%). After adjustment for sex and family background, children rated as being anxious or inattentive were more likely to remain unpartnered from age 18 to 35 years, while those rated as aggressive-oppositional or inattentive were more likely to separate and return to unpartnered status. Prosocial behaviours were consistently associated with earlier and more sustained partnership. Participants in the early-separated and delayed-or-unpartnered trajectories were also more likely to have left high school without a diploma and to have lower earnings.

**Conclusions:**

Childhood behavioural problems were associated with increased likelihood of being unpartnered and of partnership dissolution, which has implications for the psychological health and wellbeing of individuals and their families.

